# Who is the gate keeper for treatment in a fertility clinic in Germany? -baseline results of a prospective cohort study (PinK study)-

**DOI:** 10.1186/s12884-018-1690-8

**Published:** 2018-03-05

**Authors:** Eva Münster, Stephan Letzel, Jasmin Passet-Wittig, Norbert F. Schneider, Bettina Schuhrke, Rudolf Seufert, Ulrike Zier

**Affiliations:** 1 Institute of General Practice and Family Medicine, University of Bonn, Bonn, Germany; 2 Institute of Occupational, Social and Environmental Medicine, University Medical Center of the University of Mainz, Mainz, Germany; 3 Federal Institute for Population Research (BIB), Wiesbaden, Germany; 4 Protestant University of Applied Sciences of Darmstadt, Darmstadt, Germany; 5 Clinic and Polyclinic for Obstetrics and Gynaecology, University Medical Center, University of Mainz, Mainz, Germany

**Keywords:** Infertility, Fertility, First consultation, Men, Patient information, Epidemiology, Public health, Germany

## Abstract

**Background:**

It is estimated that 5-15% of all couples in industrialised nations are infertile. A perceived unfulfilled desire for a child or self-identification as infertile can lead to psychological strain and social isolation. About 53.000 women underwent assisted reproduction treatments in Germany in 2014. Little is known about the first medical consultation and patient needs prior to the first visit in a fertility clinic in Germany. The baseline survey of the prospective cohort study on couples undergoing fertility treatment in Germany (PinK Study) provides first results on this topic for Germany.

**Methods:**

The baseline survey was conducted between 2012 and 2013. Self-administered questionnaires were handed out to patients of six fertility clinics at the beginning of treatment by clinic staff. At a participation rate of 31.0%, we were able to analyse data on 323 women and 242 men.

**Results:**

92.6% of the women had their initial medical consultation on their unfulfilled desire for a child with a gynaecologist. After the urologist (44.2%), the general practitioner (12.0%) was the second most approached initial contact person for men. 36.4% of all men had no medical consultation on the unfulfilled desire for a child before visiting a fertility clinic. 46.9% of the respondents expressed the wish that the conversation about infertility should be initiated by a physician. Prior to their first visit to a fertility clinic, 11.2% of the men and 24.8% of the women were informed by a physician that infertility treatment can cause emotional strain.

**Conclusion:**

While almost all women consult a gynaecologist prior to the first visit in a fertility centre, one out of three men do not consult any physician at that stage. For the remaining group of men, urologists and general practitioners are the most important contact persons. Gender-specific health care needs are evident. In order to close the health care gap for men in Germany, more opportunities for discreet access to consultation should be offered. Due to its low threshold and family-oriented approach, general practice could make an important contribution to this effect.

## Background

An unfulfilled desire for a child and the self-identification as infertile can create an emotionally extreme situation for women and men as well as the whole couple [1–4]. Even lower levels of strain can lead to increased personal and relationship stress as well as social withdrawal [1, 5, 6]. Furthermore it has been reported that during fertility treatment more than 50% of the patients experience some degree of emotional distress and about 23% discontinue prematurely because of the perceived burden of treatment [7].

Although some have argued against the possibility of an objective definition of infertility that would ignore the self-perceptions of patients [8], according to the World Health Organization, infertility is to be understood as the failure of a couple to conceive after 12 months or more of regular unprotected sexual intercourse [9].

In accordance with this latter definition, it is estimated that between 5% and 15% of all couples in industrialised nations face current infertility [10]. There is a lack of reliable current data on the prevalence of infertility in Germany. A telephone survey in 1998 found a point prevalence of the phenomenon of 3% and a lifetime prevalence of 15% in women aged between 20 and 44 years [11]. Using data from the IMS Disease Analyzer database containing information on patients of 2500 practices of general practitioners and specialists, Ziller et al. [12] estimated that 8.9% of all women aged 18-45 consulted a physician to get information, testing or treatment concerning infertility between 2006 and 2010. However, assisted reproduction technology (ART) treatments are on the rise [13]. In Germany, 57.998 women have undergone in vitro fertilisation (IVF) or intracytoplasmic sperm injection (ICSI) in 2015 [14]. In Germany, 530.240 women have undergone these kinds of treatments between 1997 and 2014 [15] and 233.749 babies were born as a result of them within the same time frame [14]. It is estimated that worldwide, about 5 million babies have been born due to ART treatments since 1978 [16]. At 6.1%, Germany’s neighbouring country Denmark had the highest share of births associated with ART treatment in Europe in 2012 [13].

Access to ART treatments as well as the requirement of comprehensive patient information, treatment indications and admission requirements for physicians are regulated by professional guidelines in Germany [17]. Physicians accredited by statutory health insurance may administer ART treatments to patients only after referral by an independent family doctor or specialist. This referral to a fertility clinic requires that both partners have to have been informed on the medical, psychological and social aspects of ART and insemination by the referrer. Furthermore, the indicating diagnosis of infertility has to be a confirmed one [18].

Health effects of the unfulfilled desire for a child as well as of an ART treatment are often underestimated [19]. ART treatments pose health risks especially to women. One of the dreaded side effects is the ovarian hyperstimulation syndrome (OHSS) which can lead to severe health impairments. Another health risk is posed by the increased rate of multiple and premature births. The treatment itself and the involved periods of waiting often cause strong emotional strain to patients [20–23]. The gate keeper to the fertility clinic thus takes over a significant role: He/she has to be selective in offering ART treatments and is required to inform about the possible risks of such treatments before he/she gives the referral.

Regarding Germany, success rates of ART treatments are reported to be lower than 20% per treatment cycle [13, 15, 22]. However, the prospect of success of these treatments is overestimated by 2/3rds of the German population [24].

In addition to ensuring the comprehensive education of the general population on infertility and its risk factors, a further aim of public health should be the reduction of psychosocial stress situations by supporting infertile couples by means of consultations and counselling early on. Therefore, the first medical consultations on infertility as well as the decision on whether a referral to a fertility clinic is made are highly significant.

Despite the relevance of unfulfilled desires for a child for public health, there is a lack of comprehensive medical survey data on the subject in Germany. From our point of view, the following questions are of particular interest: Which type of doctor conducts the first medical consultation with women and men with an unfulfilled desire for a child? Does the patient or the doctor initiate the first consultation and which is preferred by the patient? Are patients being informed about the potential emotional strain during ART treatments before their first visit to a fertility clinic?

Now for the first time, these aspects can be examined in the interdisciplinary survey of couples in infertility treatment (PinK study).

## Methods

A methodological report of the PinK study has already been published [25]. The PinK study is a prospective survey on patients at all seven sites in six cities of the five fertility clinics in Rhineland-Palatinate and the fertility clinic in the capital city of Hesse (Wiesbaden). Using a standardised gender-specific questionnaire in German and Turkish language to be completed by each partner separately at home and returned by return envelope, the study’s anonymous baseline survey was conducted between July 2012 and May 2013. The study documents were handed to couples by staff members of fertility clinics if the following eligibility criteria were met:

1. At least one of the partners had their primary residence in Germany.
2. At least one of the partners had to have sufficient knowledge of the German or Turkish language to fill out the questionnaire.
3. The couple was just beginning their treatment at the fertility clinic. The study documents were handed over during the informed consent discussion for the first treatment (not necessarily ART) at the clinic.

Patients or couples, who had already been in treatment at the same fertility clinic and were for example changing the type of treatment they were receiving, were excluded from the survey.

At a participation rate of 31.0%, data on 565 subjects (323 women and 242 men) could be analysed. In 234 patient couples, both partners participated in the survey.

### Variables

To describe the overall study population as well as gender subgroups, the socio-demographic variables age, marital status, parity, health insurance type and education level were used. Educational attainment was measured by “International Standard Classification of Education (ISCED)” level and categorized into “low”, “medium” and “high” [25].

The main findings are based on the following questions:

1. Before your very first visit to a fertility clinic, which doctor did you first speak to about not getting pregnant?
2. Did the doctor initiate the conversation about the topic “not getting pregnant”?
3. Do you think that doctors should initiate a conversation about that topic on their own accord? Have you previously been informed by a doctor that treatment at a fertility clinic can possibly be emotionally stressful for you? If so, when?

Missing values (“not specified”) were assigned to the reference category (“ref.”) if they accounted for less than 2% of cases. Otherwise they were assigned their own category.

### Statistical methods

Group differences between genders were evaluated by means of Chi-Squared tests and, in case of the non-linear continuous age variable, the Mann-Whitney U test using statistical software SPSS 22. Statistical significance was defined as α < 0.05.

## Results

The overall study population of 565 subjects is 34.3 ± 5.3 years old on average (median: 34) with women being significantly younger at 32.8 ± 4.4 years of age (min.: 22, max.: 44, median: 32) than men at 36.2 ± 5.9 years of age (min.: 23, max.: 62, median: 35). 85.0% of the participants are married. 85.0% is also the number of those who have no children. 44.2% are assigned to the category “medium level of education” (see Table 1). At 87.9%, women do significantly (*p* = 0.01) more often have statutory health insurance instead of private insurance than men with 80.2%.

**Table 1:** Socio-economic characteristics of the study population of the PinK study, stratified by gender

	Total		Men		Women		*p*-value
	*n* = 565	%	*n* = 242	%	*n* = 323	%	
Age groups (in years)							< 0.001
< 35 (ref.)	317	56.1	108	44.6	209	64.7	
≥ 35	248	43.9	134	55.4	114	35.3	
Marital status							0.078
Married (ref.)	480	85.0	213	88.0	267	82.7	
Not married	85	15.0	29	12.0	56	17.3	
Parity							0.888
Childless (ref.)	480	85.0	205	84.7	275	85.1	
One child or more	85	15.0	37	15.3	48	14.9	
Health Insurance Status							0.011
Statutory (ref.)	478	84.6	194	80.2	284	87.9	
Private	87	15.4	48	19.8	39	12.1	
Education level (ISCED)							0.059
Low	20	3.5	11	4.5	9	2.8	
Medium (ref.)	250	44.2	94	38.8	156	48.3	
High	266	47.1	127	52.5	139	43.0	
Not specified	29	5.1	10	4.1	19	5.9	

Information on outcomes concerning the first medical consultation is shown in Table 2. More than one third of the men stated that they had not consulted a doctor prior to their first visit at a fertility clinic. The same was only the case for 2.5% of the women. 92.6% of the female subjects had had their first consultation about not getting pregnant with their gynaecologist, 44.2% of the male subjects had spoken to an urologist. 6.9% of all subjects had chosen their general practitioner (GP) as their first contact person. The GP was chosen significantly more often by men (12.0%) than by women (3.1%). 5.8% of men named their partner’s gynaecologist as their first contact person. The category “other” includes endocrinologists, haematologists and andrologists.

**Table 2:** Type of first physician contacted by the patient, reported initiation of conversation by physician and patient attitude towards initiation of conversation by physician

	Total		Men		Women		*p*-value
	*n* = 565	%	*n* = 242	%	*n* = 323	%	
First medical consultation with…							< 0.001
Gynaecologist	313	55.4	14	5.8	299	92.6	
General Practitioner	39	6.9	29	12.0	10	3.1	
Urologist	109	19.3	107	44.2	2	0.6	
Other (Ref.)	8	1.4	4	1.7	4	1.2	
No first consultation	96	17.0	88	36.4	8	2.5	
Doctor initiated conversation…							< 0.001
Yes	100	17.7	25	10.3	75	23.2	
No	354	62.7	121	50.0	233	72.1	
No first consultation	96	17.0	88	36.4	8	2.5	
Not specified	15	2.7	8	3.3	7	2.2	
Doctor should initiate conversation…							< 0.001
Yes	265	46.9	82	33.9	183	56.7	
No	141	25.0	69	28.5	72	22.3	
I don’t know	159	28.1	91	37.6	68	21.2	

Approximately one out of four women and one out of ten men reported that the physician first contacted by them had initiated the conversation about the pair’s potential infertility on his own account. 46.9% of all subjects, more than half of all women and every third men, advocated that doctors should address the topic themselves. 81.0% of those who were in fact approached by the doctor stated that doctors should choose this course of action.

**Fig. 1:**
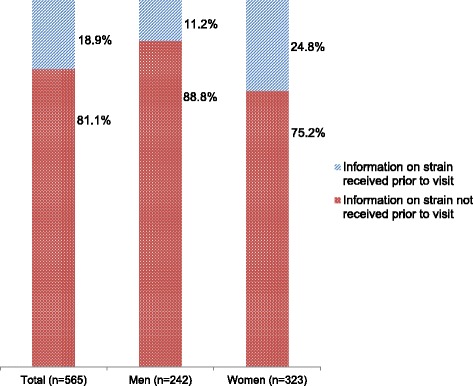
Receiving information on possible emotional strain prior the visit in a fertility clinic, PinK study (*p* < 0.001)

Less than one in 5 participants (every 9th man and every 4th woman) was informed by a doctor prior to their visit to a fertility clinic that an ART treatment could be emotionally straining (see Fig. 1).

## Discussion

The written PinK survey of 565 patients in fertility clinics in the south-west of Germany allows insight into the health service pathways of patients at the beginning of infertility treatment in Germany for the first time. It shows that medical needs and utilisation of services differ in men and women with an unfulfilled desire for a child. While nine out of ten women had spoken to their gynaecologists about the unfulfilled desire for a child, every third man had not spoken about the problem to any physician at all prior to their first visit to a fertility clinic. After the urologist, the GP was the most important contact person regarding the unfulfilled desire for a child for men (12.0%), followed by their partner’s gynaecologist. Other specialists such as endocrinologists, haematologists and andrologists were only sporadically. None of the participants stated that they had consulted a specialist for skin and sexually transmitted diseases. Only about every 4th woman and every 10th man had had the conversation about a possible infertility being initiated by their firstly contacted doctor. The patients’ needs differed from this reality: Every third man and even every second women wished that the physician would initiate a conversation about the topic. Additionally, approximately every third man and every fifth woman were indecisive and at least did not reject the idea of a consultation initiated by the doctor. It must be emphasised that only every 9th man and every 4th woman was informed that treatments at a fertility clinic could be emotionally straining, despite binding guidelines for statutory health insurance-accredited physicians requiring that patients have to be briefed on medical, psychological and social aspects of fertility treatment before their referral to a fertility clinic [18].

When interpreting the results, the reader needs to be aware that only patients of selected fertility clinics were surveyed. These patients only represent the part of the group of individuals with an unfulfilled desire for a child that is using medical services and is potentially accepting health risks due to reproductive treatment. But it is known that 10-50% of all couples struggling with an unfulfilled desire for a child do not seek medical help [10, 11, 26] and in some other cases sufficient medical support can be rendered without the need for a referral to a fertility clinic, for example through monitoring of the menstrual cycle or hormonal stimulation therapy. The PinK study does not give an insight into the medical care received by those infertile couples that do not start treatment at a fertility clinic. It also does not give any information about the selection mechanisms on the way to a fertility clinic or on the factors that decide whether a couple seeks treatment at a fertility clinic.

The following limitations apply to our results:

Selection bias could have occurred at a participation rate of 31%. A test of the study population’s representativeness of all couples at the beginning of infertility treatment was not possible due to the lack data on socio-economic variables for the latter population, e.g. in the German IVF register [14, 15]. It is notable that only very few participants (3.5%) have a “low education level”. It is possible that patients with low income utilise services at fertility clinics less frequently due to the often required financial commitment or inadequate information about treatment options. Future research will have to determine whether access and utilisation of ART treatments are unequally distributed by income in the German health care system. Also, bias in the patient selection by staff members of the fertility clinics handing out the study documents cannot be ruled out. However, this bias should have been counteracted against by the use of standardised inclusion criteria and frequent preventative communication between the coordinating study centre and staff at the fertility clinics.

It is lastly possible that an information bias, especially due to socially desirable response behaviour, may have occurred, although socially desirable response behaviour should have been prevented through ensuring full anonymity in the survey. Information bias was also mitigated by the standardisation of the survey instrument and the preceding pilot study [25].

A health care gap for men can be identified since approximately one third of male participants did not have any medical conversation about the unfulfilled desire for a child outside the fertility clinic. This result is supported by a recently conducted survey of childless persons in Germany, showing that involuntarily childless men have undergone medical examination for infertility less than half as often as women across all age groups (for example 12% men vs. 46% women aged 40 to 50 years) [26]. Need for counselling for infertile men is indicated since it is proven that male causes of infertility are involved in about 50% of infertile couples in Europe and the US [27] and that men also experience grief and elevated levels of infertility-related anxiety before and during infertility treatment [2, 28]. On the one hand, it is known that men show a lower general health care utilization than women [29, 30]. On the other hand, there seems to be a lack of a dedicated medical contact person for fertility problems for men as opposed to for women. Medical check-ups at the gynaecologist might lead to a more frequent consideration of a women’s fertility and wish for a child.. Men are lacking a comparable, low threshold contact in the health care system. As the GP is a physician that men might be comparatively regularly in contact with for a number of preventative as well as curative measures, he could be an important and feasible first contact person for men with regard to fertility questions. In Great Britain, the GP is officially recommended as the first medical contact person concerning such matters [31]. There, the majority of patients agrees with the GP’s involvement, only 3% reject it [32].

Considering that formal support is especially important for infertile men as they are less likely than women to seek informal support in their social network and are more prone to withdrawal [2], the health care gap for infertile men in Germany urgently needs to be closed. Due to the low threshold, family-oriented approach of GPs and the trust placed in them by men and women alike, they might be able to discuss fertility problems and give medical information on the possibilities of possible treatments. They could also help to lower unrealistically high expectations regarding the prospect of reproductive medical treatments and offer advice regarding alternatives.

A part of the patient collective would like to be approached by their doctor regarding an unfulfilled desire for a child. But in a survey of 25 GPs in a German city for example, only few GPs reported having in fact initiated a conversation about fertility problems [33]. The new findings reported here should motivate doctors to address the topic of fertility and according problems.

It would definitely be of advantage and is also a legal obligation for statutory co-payment for ART treatment in Germany that doctors referring to a fertility clinic inform the patients about treatments, alternatives and possible strain [31, 34]. An earlier study has already suggested that patients wish for more information on the possible psychological strain during the treatment [35]. This information need is further supported by results from a study on childless men and women in Germany that found that far more men and women have heard about treatment measures (e.g. IVF, insemination and sperm or egg donation) than of psychosocial counselling and psychotherapy for persons with an unfulfilled desire for a child. The degree of familiarity with the latter services amounted to only 45%, respectively 46% among men and 53%, respectively 57% among women [26].

## Conclusions

Results from the PinK survey show for the first time that many different groups of physicians are involved in their patients’ infertility problems in Germany and that health care services need to be improved, especially with regard to men.

Further nationwide representative surveys are necessary to fully analyse the medical care situation of all people with an unfulfilled desire for a child and to comprehensively conclude the possible courses of action.

Access to health care and consultation in case of an unfulfilled desire for a child should be obstacle-free and provided in a timely manner. This is equally important for men and for women as infertility treatment always affects the infertile couple and is not limited to one of the partners. Whereas women usually consult the gynaecologist in the case of an unfulfilled desire for a child, a health care gap still needs to be closed for men in Germany. The increased involvement of the GP could be helpful here as he is usually timely available and offers a low threshold, discreet access to an initial consultation and thereby to an introduction to the topic especially for men.

## Abbreviations

PinK: Paare in Kinderwunschtherapie [*German:* Couples in Infertility Treatment]
ART: Assisted Reproduction Technologies
ICSI: Intracytoplasmic Sperm Injection
IVF: In Vitro Fertilisation
OHSS: Ovarian Hyperstimulation Syndrome
ISCED: International Standard Classification of Education
GP: General Practitioner
